# Erythropoietin promotes the growth of pituitary adenomas by enhancing angiogenesis

**DOI:** 10.3892/ijo.2011.1261

**Published:** 2011-11-11

**Authors:** JINSHENG YANG, ZHENG XIAO, TAO LI, XUANMIN GU, BO FAN

**Affiliations:** Department of Neurosurgery, The First Affiliated Hospital of Henan University of Science and Technology, Luoyang, P.R. China

**Keywords:** pituitary adenoma, erythropoietin, angiogenesis

## Abstract

rhEPO is frequently used in clinical practice to treat anemia. However, recently rhEPO has been reported to accelerate tumor growth, progression and metastasis. Many pituitary adenoma patients, particularly those with macroprolactinomas, tend to have anemia and may need rhEPO therapy. To date, whether rhEPO has deleterious effects on pituitary adenomas has not been defined. Here we demonstrated for the first time that human pituitary adenomas are EPOR negative tumors and rhEPO accelerated the tumor growth of MMQ pituitary adenoma xenografts via enhancement of angiogenesis *in vivo*, whereas rhEPO displayed no direct effect on MMQ cells *in vitro*. Our mechanistic study showed that rhEPO administration increased phosphorylation of JAK2, STAT3 and VEGF expression in human umbilical vein endothelial cells (HUVECs) *in vitro* and in MMQ cell xenografts *in vivo*. Furthermore, VEGF inhibitor attenuated rhEPO induced angiogenesis and delayed tumor growth in MMQ pituitary adenoma xenografts *in vivo*. JAK2 inhibitor AG490 attenuated EPO induced HUVECs proliferation, phosphorylation of JAK2, STAT3 and VEGF upregulation *in vitro* and inhibited EPO induced vessel formation in Chicken chorioallantoic membrane (CAM) angiogenesis model *in vivo*. These results suggest that rhEPO administration may promote the growth of pituitary adenomas by enhancing angiogenesis through EPO-JAK2-STAT3-VEGF signaling pathway. rhEPO should be used with caution in anemia patients bearing pituitary adenoma due to its potential deleterious effects.

## Introduction

Recombinant human erythropoietin (rhEPO) has long been used to treat anemia in many clinical settings, such as kidney failure, bone marrow disease, chemotherapy and radiotherapy (1). However, recently the non-hematopoietic biological effects of erythropoietin have been reported because of ubiquitous EPOR expression in non-erythroid cells (2–6). Among these, deleterious effects of therapeutically administered rhEPO on solid tumors have received a great deal of attention.

In 2003, solid tumor patients treated with rhEPO in two large clinical trials displayed increased mortality (2,3). Since then, many clinical studies have reported increased tumor progression, tumor growth and mortality in rhEPO treated solid tumor patients (3,6–11). Furthermore, numerous basic experimental studies also confirmed these deleterious effects in many types of tumors, including renal, breast, lung, prostate, ovarian, head and neck squamous cell carcinomas (12–17). So far, whether rhEPO administration demonstrates similar adverse effect on pituitary adenoma has not been deciphered. Since many patients bearing pituitary adenomas receive conservative treatment and anemia is common in these patients, especially those with macroprolactinomas (17–19), unveiling the potential risk of rhEPO on pituitary adenomas has high clinical value in guiding clinicians who care for anemia patients with pituitary adenomas.

In the present study, we first characterized EPOR expression in different hormone secreting types of human pituitary adenomas and found no EPOR protein expression in pituitary adenomas. Next, we investigated for the first time the effect of rhEPO administration on pituitary adenomas using a nude mouse xenograft model of rat MMQ prolactin-secreting pituitary adenoma cells. Furthermore, we also explored the underlying mechanism in human umbilical vein endothelial cells (HUVECs) *in vitro* and in chicken chorioallantoic membrane (CAM) angiogenesis model *in vivo*. Our results indicate that, at least in our cases, pituitary adenomas are EPOR-negative tumors and rhEPO administration accelerates the growth of pituitary adenomas by promoting tumor angiogenesis via the EPO-JAK2-STAT3-VEGF signaling pathway.

## Patients and methods

### Patients and samples

A series of 31 pituitary adenoma samples were obtained with pathological diagnosis and informed consent. All patients (12 men, 19 women; age range 16–63, mean age 43.50±11.00) years received surgery between April 2009 and April 2011 at the Department of Neurosurgery, the First Affiliated Hospital of Henan University of Science and Technology, Luoyang, China. Of these patients, 19 had prolactinomas, four had non-functional adenomas, two had multihormonal adenomas, four had gonadotropinomas and two had GH-secreting adenomas. For each sample, one half was immediately frozen at -80°C until protein extraction, the other half was fixed with 10% formalin and embedded in paraffin for histology and immunohistochemical analysis. The study was approved by the Research Ethics Committee of Henan University of Science and Technology. Samples were made anonymous according to ethical standards.

### Cell line and nude mice

The MMQ rat prolactin-secreting tumor cell line was used in this study, because prolactinoma is the most prevalent hormone-secreting type of pituitary adenomas and there is no mature human pituitary adenoma cell line to date (20). The MMQ rat prolactin-secreting tumor cell line was from ATCC and maintained in F12 culture medium supplemented with 5% fetal bovine serum (FBS), 10% horse serum, penicillin (100 μg/ml) and streptomycin (100 μg/ml) in a humidified incubator (37°C, 5% carbon dioxide). Human umbilical vein endothelial cells (HUVECs) were from cell culture center of Wuhan University and cultured in M200 culture medium supplemented with 20% fetal bovine serum (FBS), L-gultamine (2 mM) and heparin (50 μg/ml). All animal experiments were conducted in accordance with NIH guidelines and with the approval of the local animal use committee. Six-week-old nude mice were inoculated subcutaneously on the hind flank with MMQ cells (1×10^6^). After two weeks, xenograft tumors formed. Mice were randomized into three groups with four mice in each group. For each group, mice were injected with PBS, rhEPO (2000 U/kg, s.c., Kirin), or rhEPO plus bevacizumab (a VEGF inhibitor, 10 mg/kg, i.p., Roche) twice a week for two weeks, respectively. Tumor size was assessed by caliper measurements twice a week. Mice were sacrificed four weeks after tumor cell inoculation and tumors were excised for further analysis.

### Cell viability assay

MMQ cells were seeded on 96-well plates (2000 cells/well), and then cells were treated with various concentrations (0, 1, 5 U/ml) of rhEPO for 24, 72 and 120 h in CO_2_ incubator. HUVECs were seeded on 96-well plates (5000 cells/well), after attachment, cells were left untreated or treated with rhEPO (5 U/ml), rhEPO (5 U/ml) plus JAK2 inhibitor AG490 (20 μM/l) for 48 h. After treatment duration, the CCK-8 assay reagent was added to each well of the plate and incubated for another 1 h. Absorbance was read at 450 nm using a 96-well plate reader.

### Immunohistochemisty staining

For immunohistochemisty, tissue sections were deparaffinized and rehydrated. Antigen retrieval was accomplished with citrate buffer (pH 6.0). Endogenous peroxidase was blocked with 3% hydrogen peroxide. Subsequently, slides were blocked with 10% normal horse serum followed by the primary antibodies incubated overnight at 4°C. Mouse anti-EPOR (M20, Santa Cruz, CA) antibody, mouse anti-PCNA (Santa Cruz) and mouse anti-CD31 antibody (Pharmingen, CA) were used. Then, slides were washed and incubated with the biotinylated secondary antibody for 30 min, followed by ABC reagent (Vector Labs) and diaminobenzidine. Slides were counterstained with hematoxylin and dehydrated by sequential ethanol and xylene. Slides were mounted and analyzed. For microvascular density (MVD) determination, two areas of most intense neovascularization were chosen at low magnification ×100. Three random visual fields (x400) in each area of high vascularisation were recorded. The final microvessel density (MVD) was the mean value of six random visual fields of the two high vascularisation areas.

### Western blot analysis

Samples (30 μg of proteins) were electrophoresed on a 10–12% SDS-PAGE gel under reducing conditions and electroblotted onto nitrocellulose membrane. The membrane was blocked in 5% non-fat milk and incubated with primary antibodies overnight at 4°C. Primary antibodies used were anti-JAK2, anti-phospho-JAK2, anti-STAT3, anti-phospho-STAT3 (Millipore, MA), anti-ERK2, anti-phospho-ERK2, anti-AKT1, anti-phospho-AKT1 (Santa Cruz, CA), anti-VEGF (Pharmingen, CA). After washing, blots were incubated in secondary antibody (1:5000) for 1 h. ECL substrate was used for signal detection and GAPDH for internal control. The intensity of bands was quantified by densitometric analysis. Results were expressed as the ratio of intensity to that of internal control.

### Semiquantitative RT-PCR

Total RNA was isolated from cultured cells using TRIzol reagent (Invitrogen) according to the manufacturer’s protocol, complementary DNA was synthesized using reverse transcription reagent kit (Applied Biosystems). RT-PCR analysis was performed using the following primers: human JAK2 forward 5′-TTATGGACAACAGTCAAACAACAATTC-3′ and reverse 5′-CTTACTCTCGTCTCCACAAAA-3′. Human STAT3 forward 5′-CAAAACCCTCAAGAGCCAAGG-3′ and reverse 5′-TCACTCACAATGCTTCTCCGC-3′. Human VEGF forward 5′-CGAAGTGGTGAAGTTCATGGATG-3′ and reverse 5′-TTCTGTATCAGTCTTTCCTGGTGAG-3′. Human GAPDH forward 5′-GCTTTTAACTCTGGTAAAGTGG-3′ and reverse 5′-TCACGCCACAGTTTCCCGGAGG-3′. The PCR reactions were initiated with denaturation at 95°C for 5 min; followed by 30 amplification cycles at 95°C (30 sec), 58°C (30 sec) and 72°C (30 sec), then finally 72°C (10 min) and 4°C. The relative amount of gene was normalized against GAPDH mRNA.

### Chicken chorioallantoic membrane (CAM) angiogenesis model

Chicken eggs were kept in a 37°C, 60% humidity incubator for 9 days. The CAM was dropped and the window sealed with stretchy tape. Fibrous membranes diluted with rhEPO (5 U/ml), rhEPO plus AG490 (20 μM/l) or PBS were placed on the avascular area of the Chicken chorioallantoic membrane. After 3 days, the chicken chorioallantoic membrane was fixed in 4% paraformaldehyde in PBS, dissected and photographed using stereomicroscope equipped with a Digital camera for further angiogenesis analysis.

### Statistical analysis

Statistical analysis was conducted with the aid of SPSS 16.0 software. Data are expressed as mean ± SEM. Data obtained from two groups were analyzed by Student’s t-test. P-values <0.05 were considered significant.

## Results

### No presence of EPOR expression in human pituitary adenoma tissue and MMQ pituitary adenoma cells

**Figure 1:**
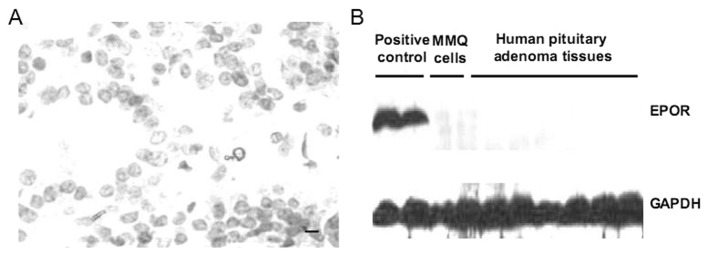
Representative images of immunoreactivity (A) and Western blot analysis (B) for EPOR in human pituitary adenomas. (A) No positive staining of EPOR in human pituitary adenomas (original magnification ×400). Scale bar, 5 μm. (B) Western blotting showed no EPOR expression in human pituitary adenomas. EPOR positive cells and tissue (HepG2 human hepatoma cells and glioma tissue) were set as positive control. Results are representative of all human pituitary adenoma samples.

Since the EPO-EPOR signaling has never been investigated in human pituitary adenomas, we first assessed the EPOR expression in 31 human pituitary adenoma samples using immunohistochemistry and Western blotting. HepG2 human hepatoma cells and glioma tissue, known EPOR-positive cells and tissue, were used as positive control. In this study, we found no EPOR protein expression in pituitary adenoma samples (Fig. 1). Moreover, similar results were also found in MMQ pituitary adenoma cells (Fig. 1). These data demonstrate that, at least in our cases, pituitary adenomas are EPOR-negative tumors and MMQ cells are EPOR-negative cells.

### No proliferative effect of rhEPO on MMQ cells in vitro

**Figure 2:**
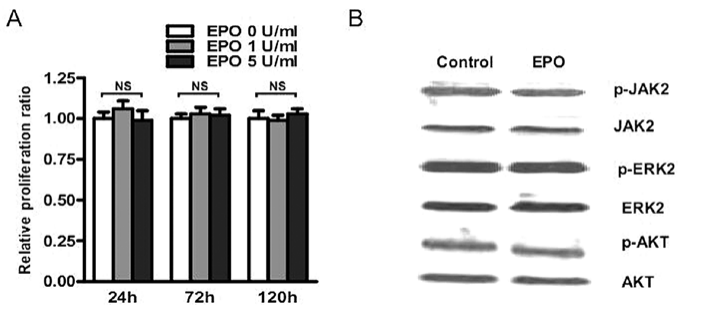
No presence of rhEPO-induced proliferation (A) and signaling pathway activation (B) in MMQ cells *in vitro*. (A) MMQ cells were cultured with indicated amount of rhEPO for indicated periods, CCK8 assay determined the cell proliferation and showed that rhEPO have no proliferative effect on MMQ cells *in vitro*. (B) MMQ cells were cultured with rhEPO (5 U/ml) for 48 h. Western blotting showed that rhEPO had not increased tyrosine phosphorylation of JAK2, ERK2 and AKT in MMQ cells. Results are mean ± SEM from three independent experiments. NS, P-values are not significant.

To further analyze the effect of rhEPO on pituitary adenomas, the potential proliferative effect of rhEPO was evaluated on MMQ cells *in vitro*. MMQ cells were starved in F12 medium containing 1% FBS for 48 h, and then co-cultured with rhEPO (0, 1, 5 U/ml) for 24, 72 and 120 h. As expected, no proliferative response was observed in MMQ cells when rhEPO were used as stimulator (Fig. 2A), indicating that rhEPO has no proliferative effect on MMQ cells *in vitro*.

### No rhEPO-induced signaling pathways were activated in MMQ cells in vitro

JAK2/STAT3, MAPK/ERK, and PI3K/AKT pathways are known to be the classic EPO-activated signaling pathways. Moreover, some of these pathways have been reported to be activated in some non-proliferative responding cancer cell lines. Therefore, we determined whether 48 h rhEPO (5 U/ml) stimulation can activate these classic pathways using Western blotting and found no increased tyrosine phosphorylation of JAK2, ERK2 and AKT in MMQ cells treated with rhEPO (Fig. 2B). These results indicate that rhEPO has no influence on these classic EPO-induced signaling pathways in MMQ cells *in vitro*.

### rhEPO administration is associated with increased tumor growth and angiogenic response *in vivo*

**Figure 3:**
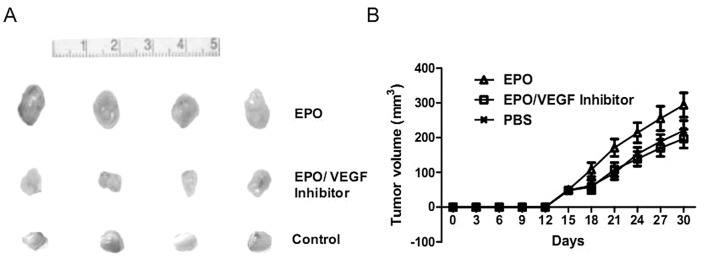
rhEPO accelerates tumor growth *in vivo*. Nude mice were inoculated subcutaneously with MMQ cells (1×10^6^) on day 0 and treated with rhEPO (2000 U/kg), rhEPO plus VEGF inhibitor (10 mg/kg) or PBS from day 15 twice per week for two weeks. (A) Picture of tumors of each group. (B) rhEPO administration significantly accelerated tumor growth, whereas VEGF inhibitor attenuated rhEPO induced tumor growth (P<0.05).

**Figure 4:**
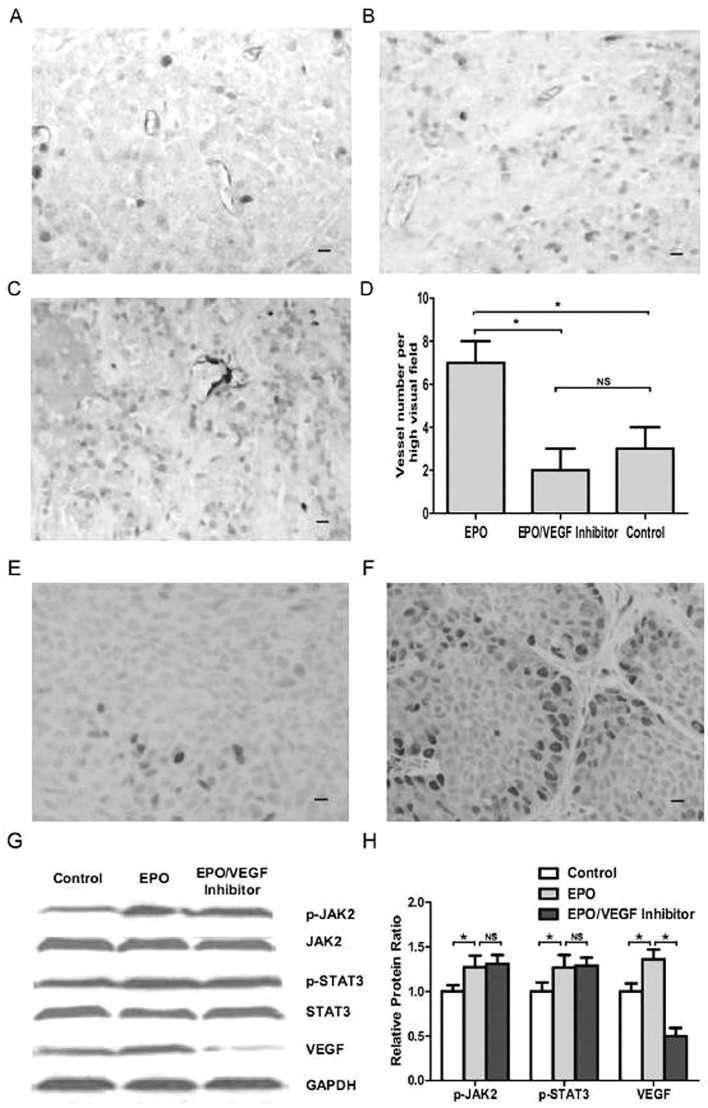
rhEPO increases tumor microvessel density *in vivo*. Representive imaging of CD31 staining in MMQ xenografts treated with rhEPO (A), PBS (B) or rhEPO plus VEGF inhibitor bevacizumab (C) respectively (original magnification ×400). Scale bar, 5 μm. (D) Tumor microvessel density analysis using CD31 immuno-reactivity showed that rhEPO increased tumor microvessel density, whereas VEGF inhibitor bevacizumab attenuated rhEPO induced angiogenesis *in vivo* (P<0.05). (E and F) Immunoreactivity for PCNA in MMQ cell xenografts. Xenografts treated with rhEPO (F) displayed increased PCNA staining compared to control group (E) (P<0.05). Scale bar, 5 μm. (G and H) Western blotting showed that rhEPO increased phosphorylation of JAK2, STAT3 and VEGF protein levels in MMQ cell xenografts, whereas VEGF inhibitor bevacizumab attenuated rhEPO-induced VEGF upregulation (P<0.05). ^*^P<0.05. NS, P-values are not significant.

As rhEPO demonstrated no direct effect on MMQ cells *in vitro*, we further examined whether rhEPO regulate tumor growth of pituitary adenomas *in vivo* using a nude mouse xenograft model of MMQ prolactin-secreting pituitary adenoma cells. Contrary to our *in vitro* results, we found that a two-week rhEPO administration (2000 U/kg, twice per week) significantly accelerated tumor growth (Fig. 3). Because rhEPO has been shown to promote proliferation of human endothelial cells and cerebral arteries express EPOR, in seeking the underlying mechanism of the *in vivo* rhEPO-induced tumor growth acceleration, we evaluated the microvessel density and tumor cell proliferation using immunohistochemisty staining of anti-CD31 and anti-PCNA antibodies and found significantly higher microvessel density (P<0.05, Fig. 4A–D) and increased tumor cell proliferation (P<0.05, Fig. 4E and F) in rhEPO treated xenograft tumors than control ones. Moreover, immunoreactivity for PCNA in EPO treated xenografts showed that tumor cells in peri-vessel areas displayed higher proliferation, which was indicated as increased PCNA staining (Fig. 4F). These results suggest a key role of angiogenesis in the rhEPO induced tumor growth acceleration in MMQ cell xenografts.

### Increased VEGF expression and phosphorylation of JAK2 in rhEPO treated MMQ cell xenograft tumors

Since rhEPO promotes angiogenesis in MMQ cell xenografts *in vivo* without direct effect on MMQ cells *in vitro*, and VEGF has been reported to play a key role in angiogenesis, in order to characterize signaling pathways involved in rhEPO induced *in vivo* tumor growth acceleration, Western blot analysis was carried out to measure VEGF expression of rhEPO treated xenografts. Being classic EPO proangiogenic signaling pathway, phosphorylation of JAK2 was also evaluated (21). We found that significant enhanced VEGF expression and phosphorylation of JAK2 in rhEPO treated xenograft tumors (P<0.05, Fig. 4G and H). Based on these observation, we hypothesized that rhEPO induced VEGF signaling may play a key role in its angiogenic effect. As STAT3 has been reported to be required for EPO induced VEGF upregulation (22), we further explored this issue and found significant increased phosphorylation of STAT3 in rhEPO treated xenograft tumors (P<0.05, Fig. 4G and H). The data demonstrate that the EPO-JAK2-STAT3-VEGF signaling axis may be involved in rhEPO induced angiogenesis.

### VEGF blockade inhibits rhEPO induced xenograft tumor growth and angiogenesis

To further verify the vital role of angiogenesis in rhEPO induced tumor growth of pituitary adenoma cell xenografts, we administered rhEPO (2000 U/kg) plus bevacizumab (10 mg/kg), a VEGF inhibitor, to nude mice bearing xenograft tumors. After a two-week administration, the bevacizumab administration significantly attenuated EPO-dependent tumor growth and angiogenesis (P<0.05, Figs. 3 and 4). These observations further confirmed the key proangiogenic role of rhEPO in its *in vivo* tumor growth acceleration.

### JAK2 inhibitor AG490 attenuated EPO-induced HUVEC survival, VEGF upregulation and phosphorylation of JAK2 and STAT3

**Figure 5:**
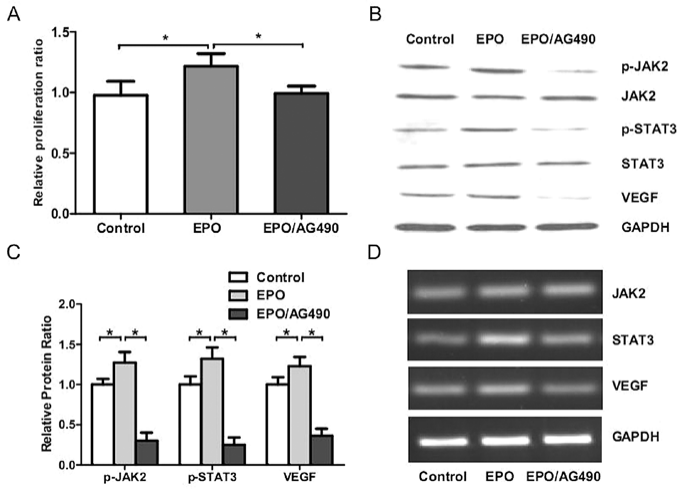
rhEPO promotes the proliferation and activates the JAK2-STAT3-VEGF signal pathway of HUVECs *in vitro*, whereas JAK2 inhibitor AG490 attenuates these above rhEPO induced effects. HUVECs were cultured without or with rhEPO (5 U/ml), rhEPO (5 U/ml) plus AG490 (20 μM/l) for 48 h and then CCK8 assay, Western blot analysis and RT-PCR were performed. (A) CCK8 assay showed that rhEPO promoted the proliferation of HUVECs *in vitro* and AG490 attenuated rhEPO induced proliferative effect (P<0.05). (B and C) Western blot analysis showed that rhEPO increased protein levels of p-JAK2, p-STAT3 and VEGF in HUVECs *in vitro* and AG490 attenuated rhEPO induced signaling activation (P<0.05). (D) RT-PCR showed that rhEPO increased mRNA levels of JAK2, STAT3 and VEGF in HUVECs *in vitro*, whereas AG490 attenuated these rhEPO induced effects except JAK2 mRNA upregulation (P<0.05). Results are mean ± SEM from three independent experiments. ^*^P<0.05.

To further verify the involvement of EPO-JAK2-STAT3-VEGF signaling axis in rhEPO-induced angiogenesis, we cultured HUVECs with rhEPO (5 U/ml), rhEPO (5 U/ml) plus AG490 (20 μM/l) or low-serum medium for 48 h, and then CCK8 assay, Western blot analysis and RT-PCR were performed. We found that rhEPO significantly promoted the proliferation (P<0.05, Fig. 5A) and activated JAK2-STAT3-VEGF signaling in HUVECs (P<0.05, Fig. 5B–D). Moreover, AG490 significantly inhibited these EPO-induced effects *in vitro* (P<0.05, Fig. 5). These results further confirmed the vital role of EPO-JAK2-STAT3-VEGF signaling axis in rhEPO-induced angiogenesis.

### AG490 inhibits EPO-induced angiogenesis in the chicken chorioallantoic membrane

**Figure 6:**
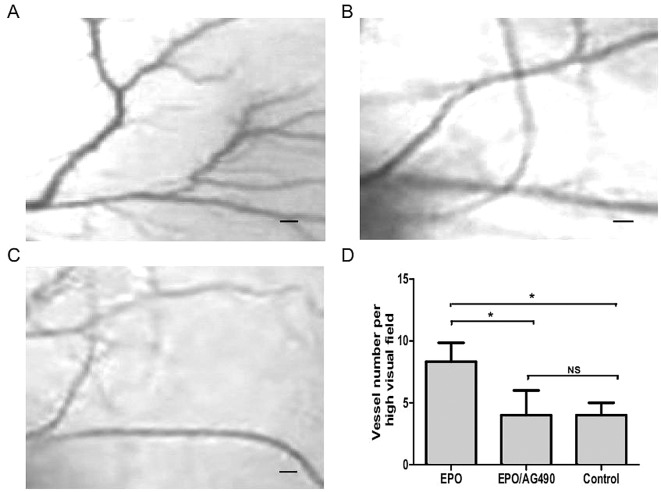
AG490 inhibits EPO-induced angiogenesis of the CAM. Fibrous membranes diluted with EPO, EPO plus AG490 or PBS were placed on CAMs from 9-day-old chick embryos. Treated areas were photographed 72 h after treatment. There was an increase in the CAM of capillary network induced by 5 U/ml EPO (A), in comparison to that induced by PBS (B) (P<0.05). This increased capillary network was inhibited by the addition of AG490 (20 μM/l) during the co-culture (C) (P<0.05). Scale bar, 1 mm. (D) Total number of vessels of the CAM in different group. Results are mean ± SEM from three independent experiments. ^*^P<0.05. NS, P-values are not significant.

To further demonstrate the proangiogenic role of rhEPO more intuitively, we investigated its effect on neovascularisation in the developing chorioallantoic membrane (CAM) of the chicken embryo. As a trigger for angiogenesis, fibrous membrane diluted with rhEPO (5 U/ml) or PBS were placed on the CAM of chicken embryos. After 72 h, vessel formation was significantly increased in EPO treated CAM compared to the control (P<0.05, Fig. 6). As shown in Fig. 6, in contrast, when rhEPO (5 U/ml) plus AG490 (20 μM/l) were further incubated on the CAM for 72 h, AG490 significantly attenuated the EPO induced vessel formation (P<0.05).

## Discussion

In this study, for the first time, we present the evidence that pituitary adenomas are EPOR-negative tumors. In addition, rhEPO accelerates pituitary tumor growth in a nude mouse xenograft model of MMQ pituitary adenoma cells accompanied with increased microvessel density and upregulation of JAK2-STAT3-VEGF signaling, whereas rhEPO displays no direct effect on MMQ cells *in vitro*. Furthermore, EPO promotes HUVECs proliferation and upregulates phosphorylation of JAK2 and STAT3 and VEGF expression, whereas JAK2 inhibitor AG490 significantly attenuated the EPO induced proliferation in HUVECs *in vitro* and vessel formation in CAM *in vivo*. Our results suggest that rhEPO may exert its *in vivo* proliferation effect via enhancement of angiogenesis in pituitary adenomas through EPO-JAK2-STAT3-VEGF signal pathway.

In the present study, a two-week rhEPO administration resulted in accelerated tumor growth of MMQ cell xenografts, suggesting that rhEPO administration can accelerate the tumor growth of EPOR negative pituitary adenomas. rhEPO has to date been described to stimulate tumor growth though both *in vitro* direct and *in vivo* indirect effects (23). Specific *in vitro* growth response to rhEPO has been reported in RCC, breast, prostate, lung and HNSCC cell lines (14,15,23–25). The direct proliferative effect of rhEPO seems to be EPOR-dependent and tumor cell specific, because previous studies have shown that growth response to rhEPO is only on those cells expressing cell-surface EPOR (23,24). Moreover, cells from different organs, even multiple cell lines derived from the same organ, display different growth response to exogenous EPO (23). These discrepancies may be due to different cell lines, different culture conditions or different assay procedures. In the present study, the lack of growth response and signal transduction to rhEPO in MMQ cells *in vitro* excluded the possibility of the direct proliferative effect of rhEPO on MMQ cells. This is consistent with previous experiments suggesting no proliferative effect of rhEPO on EPOR negative cells (26).

Besides direct proliferative effect on tumor cells, rhEPO has also been suggested as a pro-angiogenic factor, which indirectly promotes tumor growth via angiogenesis (21,27). Because EPOR has been confirmed in various endothelial cells, especially including brain capillary endothelial cells, rhEPO can directly stimulate angiogenesis of both EPOR positive and negative tumors, which in turn provide a growth advantage to tumor cells *in vivo* (21,26,28,29). Previous studies have documented that rhEPO can not only increase endothelial cell proliferation but also enhance the number of circulating endothelial cells and endothelial precursor cells (21,26). Furthermore, it is noteworthy that rhEPO can stimulate cerebral angiogenesis *in vivo* and promote capillary tube formation of cerebral endothelial cells by inducing vascular endothelial growth factor (VEGF), all of which provide evidence for the proangiogenic properties of rhEPO (28,30,31). Based on the aforementioned, we hypothesized that rhEPO may indirectly promote tumor growth by enhancing angiogenesis. In order to further clarify this issue, immunohistochemisty of CD31 and PCNA were carried out to investigate the microvessel density and tumor proliferation of MMQ cell xenografts in both rhEPO and control groups. In supporting of our hypothesis, we found increased microvessel density and proliferation in rhEPO treated MMQ cell xenografts. Moreover, tumor cells in peri-vessel areas displayed higher proliferation. These data defined a key proangiogenic role of rhEPO in stimulating the tumor growth of MMQ cell xenografts.

The EPO-JAK2-STAT3-VEGF pathway has been well documented for its essential proangiogenic role in tumor growth acceleration (21). Previous studies have reported that EPO can activate STAT3 and STAT5 in primary cerebral vascular cells (21,28,29). Furthermore, the deletion of the endogenous Epo-EPOR system in nonhematopoietic cells in mice impairs STAT3 activation, VEGF upregulation, and capillary growth (31). Our mechanistic study of MMQ cell xenografts showed that rhEPO administration increased phosphorylation of JAK2, STAT3 and VEGF expression. Moreover, VEGF blockade attenuated rhEPO induced xenograft angiogenesis and tumor growth. These results suggest a key role of the proangiogenic property of rhEPO in its tumor growth promotion of pituitary adenomas. As rhEPO has been reported to stimulate JAK2-STAT3 pathway and STAT3 is required for VEGF upregulation (22,32,33), we further verified this issue and found that JAK2 inhibitor AG490 not only attenuated EPO induced HUVECs survival, phosphorylation of STAT3 and VEGF upregulation *in vitro*, but also inhibited EPO-induced angiogenesis in the chicken chorioallantoic membrane. These results defined the EPO-JAK2-STAT3-VEGF pathway as an underlying mechanism of the rhEPO proangiogenic property, which in turn promotes tumor growth in pituitary adenomas.

In the present study, we demonstrated that, at least in our cases, pituitary adenomas are EPOR negative tumors and systematic rhEPO administration may promote tumor growth of pituitary adenomans via enhancing angiogenesis though the EPO-JAK2-STAT3-VEGF signal pathway. Based on our results, rhEPO should be used with caution in pituitary adenoma patients due to its potential detrimental proangiogenic and proliferative effect. Further research is needed to fully elucidate the complex interplay between rhEPO and pituitary adenomas, in order to identify whether rhEPO is suitable for use in anemia patients bearing pituitary adenoma.
